# Local activation of cannabinoid CB_1 _receptors in the urinary bladder reduces the inflammation-induced sensitization of bladder afferents

**DOI:** 10.1186/1744-8069-7-31

**Published:** 2011-05-09

**Authors:** Jean-Sébastien Walczak, Fernando Cervero

**Affiliations:** 1 McGill University, Anesthesia Research Unit, Faculty of Medicine, Faculty of Dentistry and Alan Edwards Center for Research on Pain, Montréal, Québec, Canada

## Abstract

**Background:**

Systemic administration of cannabinoid agonists is known to reduce pain induced by bladder inflammation and to modulate cystometric parameters *in vivo*. We have previously reported that intravesical administration of a cannabinoid agonist reduces the electrical activity of bladder afferents under normal conditions. However, the effects of local activation of bladder cannabinoid receptors on afferent activity during inflammation are unknown. This study was aimed to assess the effects of intravesical administration of a cannabinoid agonist on the discharges of afferent fibers in inflamed bladders *ex vivo*. We also characterized the expression of CB_1 _receptors in the bladder and their localization and co-expression with TRPV1, a marker of nociceptive afferents.

**Results:**

Compared to untreated animals, afferent fiber activity in inflamed bladders was increased for intravesical pressures between 10 and 40 mmHg. Local treatment with a non selective cannabinoid agonist (AZ12646915) significantly reduced the afferent activity at intravesical pressures above 20 mmHg. This effect was blocked by AM251 but not by AM630 (selective for CB_1 _and CB_2 _respectively). Finally, CB_1 _was co-expressed with TRPV1 in control and inflamed bladders.

**Conclusion:**

These results demonstrate that sensitization of bladder afferents induced by inflammation is partly suppressed by intravesical activation of cannabinoid receptors, an effect that appears to be mediated by CB_1 _receptors. Also, TRPV1 positive fibers were found to co-express CB_1_, supporting the hypothesis of a direct action of the cannabinoid agonist on nociceptive afferents. Taken together, these results indicate a peripheral modulation by the cannabinoid system of bladder hypersensitivity during inflammation.

## Background

The role of the cannabinoid system in the regulation of bladder function has attracted considerable interest in the last few years [1]. Several studies have reported the presence of cannabinoid receptors CB_1 _or CB_2 _mRNA and/or protein in the bladder of rats [2], mice [3], monkeys[4] and humans[5]. The localization of CB_1 _receptors has been described in the urothelium and in nerve fiber structures of the suburothelium and the detrusor [3,6,7]. Another study did not find CB_1 _immunoreactivity in the urothelium and nerve fibers but reported the expression of CB_2 _in these structures [4]. However, most previous studies have looked only at the efferent functions of the bladder when studying the functional activity of cannabinoid receptors. For instance, electrically-evoked contractions of bladder preparations in mice are reduced by CB_1 _activation [8,9]. The release of CGRP evoked by capsaicin and ATP is also reduced by activation of CB_1 _and CB_2 _in rats [6]. And cystometric studies have shown that systemic administration of cannabinoid CB_1 _and CB_2 _agonists increases the micturition threshold and voiding interval [10-12].

During pathological conditions there is a change in the expression of cannabinoid receptors. CB_1 _mRNA transcription was found to be increased in patients with chronic bladder pain syndromes [13] and CB_2 _mRNA and protein expressions are increased in rats with bladder inflammation due to acrolein [2]. Cystometric studies have also shown a reduction by cannabinoids of bladder hyperactivity after inflammation [10,11,14]. In addition, referred hyperalgesia due to intravesical turpentine instillation is reduced after systemic administration of cannabinoid agonists [15].

Although some of these cystometric and behavioral effects of cannabinoids could involve the afferent component of the bladder, the precise contribution of afferent effects is difficult to ascertain because of the techniques used and the systemic administration of the agonists. We have previously shown that stimulation of cannabinoid receptors reduces afferent activity in an electrophysiological *ex vivo *preparation under normal conditions [3]. However, nothing is known about the responses to cannabinoid agonists of afferents from inflamed bladders. The purpose of the present study was to assess the effect of intravesical administration of a cannabinoid agonist on afferent bladder activity after an acute inflammation induced by cyclophosphamide (CYP). We used our *ex vivo *bladder nerve preparation to evaluate the effects of inflammation as well as the action of the intravesical administration of a non-selective CB_1_-CB_2 _agonist on afferent fiber activity. In another set of experiments, we challenged this agonist with selective antagonists for CB_1 _and CB_2 _receptors (AM251 and AM630). Finally, to assess if direct activation of CB_1 _receptors on bladder afferents could mediate the effect of agonists, we looked at this receptor expression in bladder tissue and its possible co-localization with TRPV1, a neuronal marker of peptidergic afferent C-fibers in mice.

## Results

### Behavior

**Figure 1:**
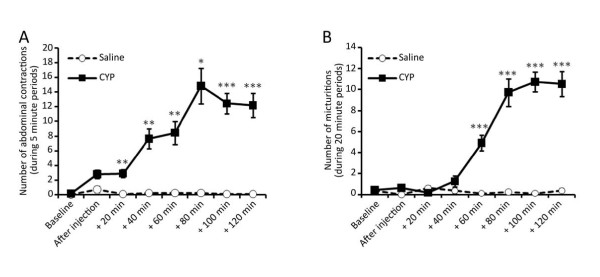
**Behavior observed after injection of cyclophosphamide.** A) Abdominal contractions observed during 5 min periods every 20 min post cyclophosphamide (CYP) or saline injection. B) Number of micturitions measured during 20 min periods every 20 min periods post CYP or saline injection. Results are mean ± SEM (Saline n = 8, CYP n = 11, **P < 0.01, ***P < 0.001, *t*-test).

Mice receiving i.p. saline produced very few abdominal contractions after the injection. Also, the number of micturitions in these control animals was less than one during each 20 minute period. Injection of cyclophosphamide (300 mg/kg) i.p. induced an increase in abdominal contractions that was statistically significant after 20 minutes post-injection. The number of abdominal contractions increased progressively reaching a plateau at 80 minutes post-injection with about 12 contractions per 5 minute period. Also, 60 minutes after injection, the number of micturitions was significantly increased in animals receiving CYP compared to those receiving saline, reaching a plateau of about 10 voids per 20 minute periods (Figure 1).

### Electrophysiology

#### -Effect of cyclophophamide treatment on bladder afferents

**Figure 2:**
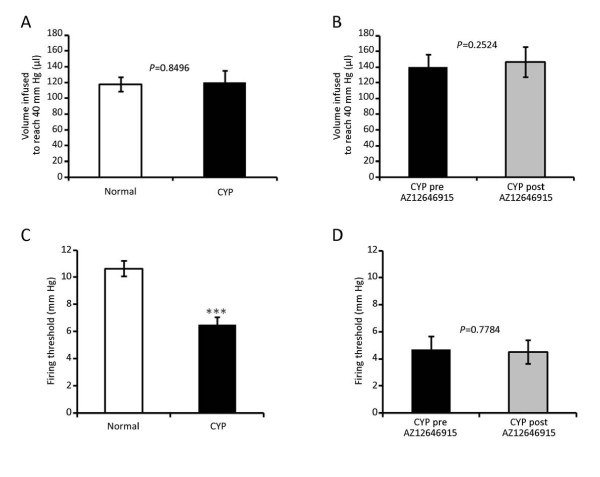
**Effects of cyclophosphamide and AZ12646915 on the bladder compliance and firing threshold of the afferent fibers.** A and B) Compliance of the bladder calculated as the volume infused to reach 40 mm Hg in normal (29 mice) and cyclophosphamide (CYP) (19 mice) pooled groups (A) and pre- versus post-administration of AZ12646915 in CYP mice (7 mice) (B). C and D) Firing threshold (mm Hg) of afferent fibers responding to bladder filling in normal (146 fibers, 52 mice) and CYP (63 fibers, 19 mice) pooled groups (C) and pre and post AZ12646915 in CYP mice (29 fibers, 7 mice). Results are mean ± SEM. Normal versus CYP analyzed with *t*-test (*** *P *< 0.001) (A and C). Pre- versus post-drug analyzed with paired *t*-test (B and D).

**Figure 3:**
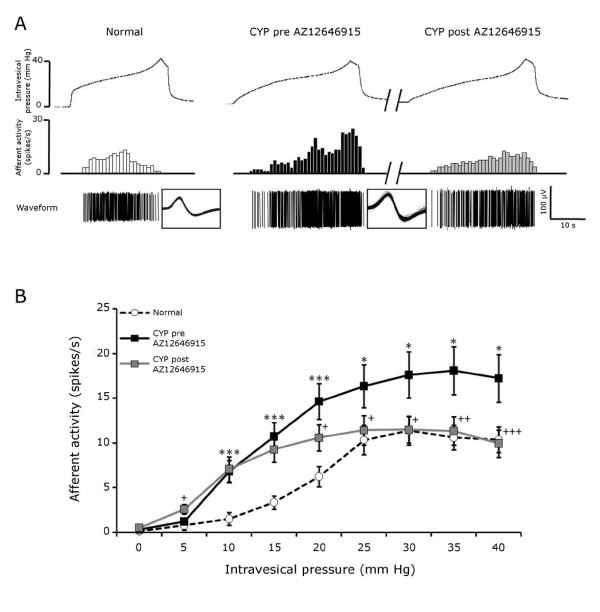
**Effects of cyclophosphamide and AZ12646915 on afferent activity in response to mechanical distension.** A) Representative traces of one fiber activity in a normal mouse (left panel) and of another fiber from an inflamed mouse (CYP) before and after the intravesical administration of the cannabinoid agonist AZ12646915 (100 μM) (middle and right panels). The superimposed waveforms of each action potential are displayed in the boxes. B) Afferent activity (spikes/s) in response to an increase of intravesical pressure for normal and inflamed (CYP) before and after administration of AZ12646915. Results are mean ± SEM, * *P *< 0.05, ***P < 0.001 for normal versus CYP (*t*-test) and + *P *< 0.05, ++ *P *< 0.01, +++ *P *< 0.001 versus CYP pre AZ12646915 (paired *t*-test). Normal: n = 32 fibers, 10 mice. CYP: n = 29 fibers, 7 mice.

Compared to normal bladders, cyclophosphamide treatment did not change bladder compliance. The volume needed to reach 40 mmHg was 117.9 ± 9.2 μl and 114.9 ± 12.8 μl for normal and inflamed bladders respectively (Figure 2A). However, the average firing threshold of afferents from inflamed bladders was significantly decreased compared to our database of thresholds from normal bladders (10.6 ± 0.56 and 6.5 ± 0.57 mmHg for normal and CYP bladders respectively) (Figure 2C). Figure 3A shows representative traces of single units responding to distention of normal (left panel) and inflamed bladder (pre-treatment, middle panel). The average activity of afferent fibers after cyclophosphamide inflammation was increased for intravesical pressures ranging from 10 to 40 mmHg. The maximal average activity was increased from about 11 to 17 spikes/s (Figure 3B).

#### -Effect of intravesical administration of a cannabinoid agonist

**Figure 4:**
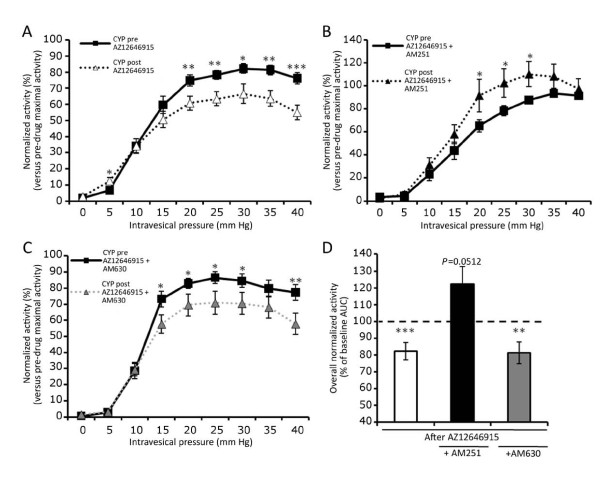
**Activity of inflamed (CYP) bladder afferent fibers, normalized to the maximal activity of pre-drug stimulation**. A) normalized activity before and after AZ12646915 alone (n = 29 fibers, 7 mice). B) normalized activity before and after AZ12646915 + AM251 (n = 19 fibers, 6 mice), C) normalized activity before and after AZ12646915 + AM630 (n = 19 fibers, 6 mice) and D) represents the percentage of the area under the curves from A, B and C. Data are mean ± SEM. Paired *t*-test was used for A, B and C and one sample *t*-test for D. * *P *< 0.05, **P < 0.01 and ***P < 0.001 versus pre-drug groups.

The bolus administration of AZ12646915 (100 μM, 100 μl) did not change bladder compliance (139.8 ± 16.2 and 146.5 ± 19.2 μl to reach 40 mmHg for pre- and post-drug respectively) or the average threshold of firing of the afferents (4.7 ± 1.0 pre-drug and 4.5 ± 0.9 mmHg post-drug) (Figure 2B and 2D). A representative recording of a single unit from an inflamed bladder before and after intravesical treatment with AZ12696915 is shown in Figure 3A (middle and right panels). The maximal activity was decreased by cannabinoid administration from 25 spikes/s to 12 spikes/s in this example. Note that most of the reduction occurred at higher intravesical pressures. The average afferent activity was significantly reduced for intravesical pressures above 20 mmHg (Figure 3 and Figure 4A). In addition, the average firing rate after AZ12646915 in inflamed bladders reached values similar to those of normal bladders for intravesical pressures ranging from 25 to 40 mmHg (Figure 3).

#### -Effects of CB_1 _and CB_2 _antagonists on the response of AZ12646915

The specificity of action of the cannabinoid agonist AZ12646915 was assessed with two antagonists: AM251 and AM630 which are selective for CB_1 _and CB_2 _respectively. The treatment with AM251 (100 μM) blocked the reduction of afferent activity observed after AZ12646915 alone (Figure 4B). Furthermore, there was an increased response of the bladder afferents for pressures going from 20 to 30 mmHg. However, this suppression of inhibition was not observed with the cannabinoid receptor CB_2 _antagonist AM630 (100 μM). The afferent activity after AZ12646915 + AM630 was reduced for intravesical pressures above 15 mmHg which is similar to the effects of the cannabinoid agonist alone (Figure 4C). The areas under the curve (AUC) for pre- and post-treatments were calculated. They showed an overall significant reduction of about 20% of afferent activity for AZ12646915 alone and AZ12646915 + AM630 while the AUC tended to be increased (*P *= 0.0512) after the treatment with AZ12646915 + AM251 (Figure 4D).

### Immunofluorescence

**Figure 5:**
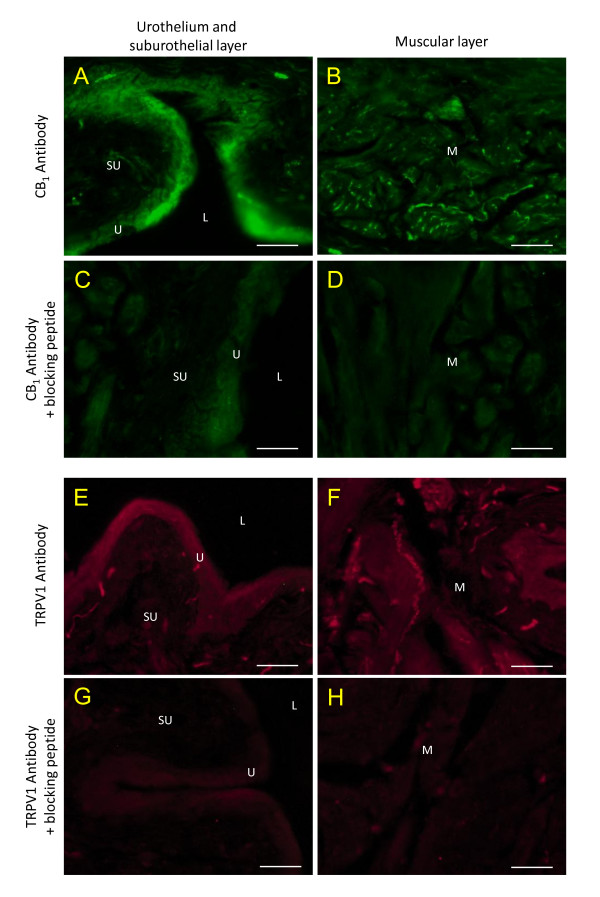
**Control for the selectivity of CB_1 _and TRPV1 receptors antibodies**. Upper panels (A-D, green) show the immunoreactivity obtained in normal bladder sections incubated with the CB_1 _antibodies alone (A, B) or with the specific blocking peptide (C, D). The lower panels (E-H, red) show the reactivity obtained with TRPV1 antibodies alone (E, F) or with the specific blocking peptide (G, H). U = urothelium, L = lumen, SU = suburothelial and M = muscular layers, scale bar = 20 μm.

**Figure 6:**
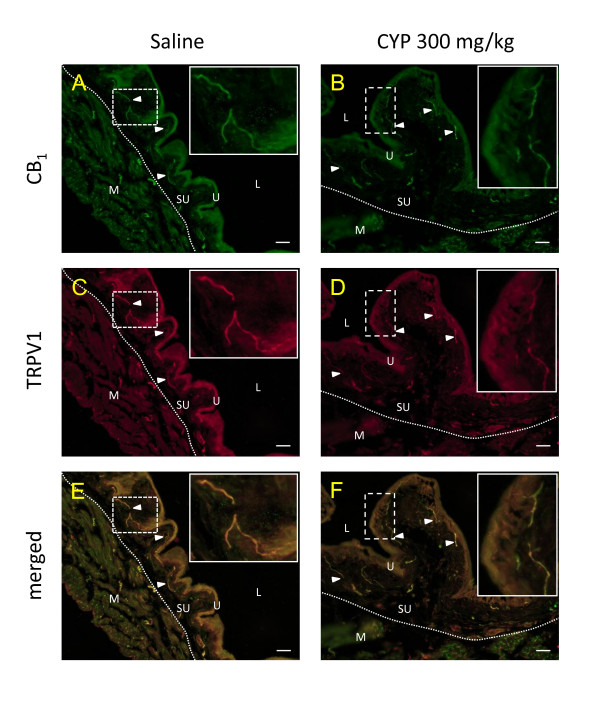
**Bladder immunoreactivity for CB_1 _receptor (A, B) and TRPV1 (C, D)**. Mice were injected with saline (control, A, C and E) or cyclophosphamide (CYP, B, D and F) 300 mg/kg two hours before tissue harvest. Arrowheads = nerve fibres which co-express CB_1 _receptors and TRPV1, U = urothelium, L = lumen, dotted line = separation between the suburothelial (SU) and the muscular (M) layers. The boxes focus on the co-expression of CB_1 _with TRPV1 in fibers of the suburothelium layer, scale bar = 20 μm.

The specificity of the antibodies was assessed with saturating concentration of the immunogen peptides specific to the targeted proteins. Under the conditions used, these blocking peptides prevented the immunofluorescence observed for CB_1 _and TRPV1 (Figure 5). CB_1 _receptor immunoreactivity was found in the urothelium and in nerve fibers structures of the suburothelium and of the muscular layers. When looking at co-staining of CB_1 _+ TRPV1, in saline and CYP treated animals, we observed co-localization in the urothelium and in nerve fiber structures from the suburothelial layer (Figure 6, arrowheads).

## Discussion

Cannabinoid agonists have analgesic properties by acting directly at the central nervous system as demonstrated by intrathecal or intracerebroventricular administrations [16-18]. However, targeting the peripheral cannabinoid system for analgesia could be of clinical value since it would eliminate the unwanted psychoactive effects. Peripheral actions of cannabinoids on somatic pain have also been described after topical administration [19], by using compounds that do not cross the blood brain barrier [20] or by genetic means using nociceptor-specific deletion of CB_1 _receptors [21]. However, there is no direct evidence of a peripheral action of cannabinoids on the sensory innervation of internal organs that could explain a purely local effect on visceral hypersensitivity. One study showed that increased activity of mesenteric afferents evoked by bradykinin could be reduced by systemic administration of a CB_2 _receptor agonist [22]. Another report, using an *in vitro *mesenteric preparation, concluded that the afferent activity induced by bradykinin or by mechanical distention was unchanged by administration of the non selective CB_1_/CB_2 _agonist WIN 55, 212-2, but reduced by a CB_1 _antagonist while the opposite effect was observed after serotonin administration [23]. As for the bladder, only indirect measurements of the effects of cannabinoids on its afferent activity have been performed using behavioral assessment [15,24] or cystometry [10,11,14]. These studies used systemic administration of cannabinoids, which did not exclude the effects of the compounds on the central nervous system. Also, since it has been shown *in vitro *that activation of cannabinoid receptors can reduce the contraction of the detrusor [8,9], the effects on motor activity could explain the results observed in the behavioral and cystometry studies. Therefore, the possible local and direct action of cannabinoids on bladder afferent activity, and hence on bladder pain, were still unknown.

Two main observations have been made in the present study: i) intravesical administration of a cannabinoid agonist reduced the sensitization of mouse bladder afferent fibers after acute inflammation; an effect that appears to be mediated through peripheral CB_1 _receptors only and ii) cannabinoid CB_1 _expressing nerve fibers co-express TRPV1 in the bladder. Therefore, the present results provide the first direct evidence that local activation of CB_1 _receptors reduce the inflammation-induced hypersensitivity of sensory afferent fibers in the bladder.

### Effects of acute cyclophosphamide inflammation on behavior

As previously reported [25,26], systemic administration of cyclophosphamide produced a substantial change in the mice behavior. All animals displayed many abdominal contractions that were almost absent in animals receiving saline solution. In addition, we noticed an important increase in micturitions up to about 10 voids per 20 minute period which is in line with the 20 to 30 voids per hour observed in another study using cyclophosphamide [27]. The average number of micturitions in the saline group was low. The reason could be that non inflamed mice urinated mostly during the habituation period (60 minutes before injection) and tended to sleep during the two consecutive hours. The clear nociceptive behavior expressed by the animals after cyclophosphamide injections allowed us to confirm that a painful inflammation was established before performing the *ex vivo *electrophysiological experiments.

### Effects of acute cyclophosphamide inflammation on bladder afferent activity

Once the bladder inflammation was established we analyzed the electrophysiological properties of its afferents. A few previous reports have described the effects of cyclophosphamide inflammation on the activity of bladder afferents. One study showed that an acute treatment with cyclophosphamide (two hours prior perfusion of rats) increased Fos immunoreactivity in regions of the spinal cord which receive afferent input from the bladder [28]. Other studies using patch clamp techniques showed an increased excitability of dissociated dorsal root ganglion neurons *in vitro *of rats having received a prolonged treatment with cyclophosphamide [29,30]. Using a preparation similar to ours, Yu and de Groat [31] have shown an increase of activity of afferent fibers in rat pelvic nerve in response to isotonic bladder distension after cyclophosphamide injection. However, this study did not look at discriminated single afferent fibers, therefore their mechanical threshold could not been assessed. Also, the increased activity observed could have been due to silent fibers that became responsive to distension after inflammation [32,33].

We did not find any significant differences in bladder compliance using the current *ex vivo *preparation. This is surprising, since cyclophosphamide treatment has been reported to change bladder compliance during *in vivo *cystometry experiments [34] or to modulate the electrical-evoked contractions of stripes of the detrusor in *ex vivo *experiments [35,36]. However, our results may be explained by the fact that in the present study the bladder preparation was disconnected from any efferent control of the detrusor. Therefore, we could ascertain that afferent responses to distension after cyclophosphamide treatment were not impaired by a change of compliance in our model.

### Effects of a cannabinoid agonist on the sensitization of bladder afferents

Intravesical administration of the non selective CB_1_-CB_2 _agonist AZ12646915 reduced the afferent activity of inflamed bladders. Interestingly, the reduction occurred for high intravesical pressures above 20 mm Hg and reached the spike rate of normal bladders for those higher pressures. These effects are in accordance with the absence of change of threshold after cannabinoid administration. Again, although the effects of cannabinoids on motility have been described through CB_1 _in mice using strips of bladders [8,9], those studies found mostly a prejunctional effect of CB_1 _activation. This effect was absent in the current *ex vivo *preparation since it is disconnected from any efferent control. In addition, we measured no change in the compliance after the treatment with AZ12646915 which support the hypothesis that the cannabinoid agonist did not act on the detrusor. Therefore, we could conclude that we observed a purely sensory effect. Also, the intravesical administration of the cannabinoid agonist could reduce a distribution to the muscular layer and prevent any effect on bladder motility.

The expression of CB_2 _receptor in the bladder has been described by others [2,6,12]. While a role of CB_2 _might be expected during inflammation, the reduction of afferent activity by a cannabinoid agonist was not modified by a pre-treatment of the CB_2 _antagonist AM630, whereas it was blocked by AM251, a CB_1 _receptor antagonist. We measured only the afferent fiber activity *ex vivo *once inflammation was established and therefore we did not evaluate any anti-inflammatory effects of the cannabinoid agonist *in vivo *or in a pre-emptive fashion. In addition, our results are in line with cystometric studies, where systemic administration of cannabinoid agonists have been shown to increase the intercontraction interval and micturition threshold measurements (which have a sensory component) in normal and inflamed rats. The same predominant role of CB_1 _versus CB_2 _was also observed with antagonists in these studies [10,11]. In contrast with these results, a recent study looked at cystometric parameters after administration of cannabinor, a novel CB_2 _agonist in normal rats [12]. The afferent components of cystometric measurements were modulated by the highest dose of this agonist. Therefore, a role for CB_2 _cannot be excluded. However, cannabinor was administered systemically and no antagonist was used to ascertain if the action of this drug selective for bladder CB_2 _receptors. A possible explanation for the role of CB_2 _observed in some cystometric studies would be via an action on the detrusor muscle. Then, since we measured solely the electrophysiological afferent activity, it could be the reason why we did not see a role for CB_2 _in this study contrary to some cystometric study.

Hayn *et al*. [6] evaluated bladder afferent activity indirectly by measuring CGRP release from dissected bladder domes stimulated with a mix of capsaicin and ATP. They observed a reduction of CGRP release after administration of a cannabinoid agonist. This reduction was absent in the presence of CB_1 _or CB_2 _antagonists. Overall CGRP release depends on afferent activity but also on the control of exocytosis of neuronal vesicles [37]. Therefore, taking our results into account, the difference of effect of cannabinoid receptors could be attributed to the fact that neuronal electric activity would be mediated by CB_1 _alone while the release of CGRP from neuronal vesicle could be modulated by both cannabinoid receptors. However, this is purely speculative especially since the expression of CB_2 _in neurons is still a matter of debate [38]. Another hypothesis to explain CB_2 _modulation of CGRP release is that it can be produced by other cell types than neurons, especially immune cells [39-41] which are known to express CB_2 _receptors [42,43].

### Expression of CB_1 _receptors with TRPV1 in the mouse urinary bladder

Since we observed a physiological effect mainly after CB_1 _receptor activation, immunofluorescence experiments were performed to better characterize the expression of this receptor. We looked at co-expression of CB_1 _protein with TRPV1, which is a marker of peptidergic nociceptors in mice [44]. As previously reported, we observed CB_1 _immunoreactivity in the urothelium as well as in nerve fiber structures of the sub-urothelium and the muscular layers [3,6,7]. We did not observed obvious differences (although not quantified) in the pattern of expression of cannabinoid and TRPV1 receptors after inflammation, which is not surprising as the observations were made only 2 hours after the induction of the inflammatory process. Unmyelinated C-fibers are present in the muscle, the suburothelium layer and close to urothelial cells [45-47] locations that correspond to those of our observations. Therefore, our electrophysiological results can be explained by a direct action of cannabinoid agonists on nociceptive fibers in the bladder modulating their inflammation-induced sensitization. In addition, it is known that TRPV1 plays a role in the bladder sensory system [48,49] and that cannabinoid receptors can modulate TRPV1 activity [50,51]. Hence, we could speculate that a possible way of modulating afferent activity could be through an interaction of CB_1 _with TRPV1 in bladder afferent fibers. The presence of CB_1 _in peptidergic fibers in the bladder is also in line with a possible neuroinflammatory regulation as previously seen with the modulation of neuropeptide release by cannabinoids [6].

In the present study, we also observed CB_1 _immunofluorescence in urothelial cells themselves as previously reported [3,5,6]. Those cells especially the umbrella cells are important player in the bladder sensory transduction processes [52]. Therefore, in addition to a direct action on nerve fibers, another explanation for the reduction of nerve activity after cannabinoid agonist administration would be indirect through the urothelial cells.

## Conclusion

We have shown that local activation of CB_1 _receptors reduces the sensitization of bladder afferent neurons during acute inflammation. In addition, the co-expression of CB_1 _with TRPV1 in nerve fibers confirms the presence of CB_1 _in peptidergic nociceptors and can therefore explain the role of cannabinoid agonists in modulating the sensitization of bladder afferents. Taken together these results support the hypothesis of a peripheral modulation by the cannabinoid system of bladder hypersensitivity after inflammation.

## Methods

### Animals

Female C57BL/6 mice weighing 18-26 g (supplied by Charles River Canada, St Constant, Qc, Canada) were used in this study. Their estrous cycle was not controlled for the following experiments. Animals were anesthetized with urethane (2 g/kg) i.p. in saline solution (0.9% NaCl) before cardiac perfusions or in order to dissect the bladder for electrophysiological studies. All experimental procedures followed the guidelines of the committee for Research and Ethical Issues of the International Association for the Study of Pain [53] and the project was approved by the Animal Care and Use Ethics Committee of McGill University. Care was taken to minimize the number of animals used and to avoid their suffering.

### Induction of inflammation

Mice received an injection of cyclophosphamide 300 mg/kg (i.p.) diluted in saline solution. This dose generates an inflammation after 2 hours post injection, as previously described [25,26]. To ensure that inflammation due to cyclophosphamide was effective, some animals were observed for their behaviors during the 2 hours preceding the dissection of the bladder for electrophysiological experiment.

### Behavior

Mice were placed 60 minutes before injection to habituate in an elevated plexiglass cage equipped with a mesh floor and a 45° angled mirror placed below to facilitate observation. To get baseline values, the behavior was observed for abdominal contractions (brief retractions of the abdomen) and the number of micturitions during the last 5 and 20 minutes of habituation respectively. A micturition episode was defined as any obvious voiding of urine. The animals received cyclophosphamide or its vehicle (saline) and the counting of abdominal contractions (for 5 minutes periods) and micturitions (for 20 minutes periods) were then repeated every 20 minutes during the 2 hours after injection. The animals were then anesthetized with urethane and prepared for the dissection of the urinary bladder.

### Electrophysiology

The mouse urinary bladder and surrounding tissues were dissected and placed in a chamber where it was continuously perfused with oxygenated (95% O_2_, 5% CO_2_) Tyrode solution pH 7.4 (content in mM: NaCl 136.9; KCl 2.7; CaCl_2 _1.8, MgCl_2 _1; NaH_2_PO_4 _0.4; NaHCO_3 _11.9 and Glucose 5.6). A fine triple-lumen canula (combined diameter 0.9 mm) was inserted into the bladder through the urethra. The canula was connected to: i) a syringe pump (Harvard Apparatus, Holliston, Massachusetts, USA) to infuse Tyrode into the bladder, ii) to a pressure transducer (P75, Hugo Sachs Elektronik - Harvard Apparatus GmbH, March-Hugstetten, Germany) to record intravesical pressure changes and iii) to an outlet tube equipped with a three way stopcock to block or release intravesical fluids from the bladder. An additional canula (MicroFil MF34G 0.1 mm ID, WPI, Sarasota, FL, USA, diameter 0.164 mm) was added along the triple-lumen canula and connected to a 1 ml syringe to allow bolus application of drugs. The urethra and the ureters were ligated to avoid leakage of fluid from the urinary bladder.

Under a microscope, a branch of the pelvic nerve arising from the urinary bladder was dissected. Nerve activity was recorded from very fine filaments teased from the pelvic nerve and sucked inside a glass suction electrode connected to an Axon Instrument head stage (AI 402 × 50 Ultra low noise differential amplifier, Axon Instrument) and an AC/DC amplifier (CyberAmp 380, Axon Instrument). Signals were amplified (250×) filtered (band-pass 10-10000 Hz) and relayed to a noise eliminator (Hum Bug, Quest Scientific, Vancouver, Canada). The electrical activity of the nerve and the intravesical pressure were digitized using a computer connected to a Micro 1401 MK II analog-to-digital interface controlled with Spike 2 (version 6.13) software (Cambridge Electronic Design, Cambridge, UK).

Mechanical stimulation of the bladder from normal or inflamed animals was performed by a slow infusion of Tyrode solution (0.1 ml/min) until the intravesical pressure reached 40 mm Hg, then the pressure was released by opening the outlet valve. Single unit discrimination was performed by using the spike sorting function of Spike2 software.

The effects of inflammation or drug administration on bladder compliance were evaluated by calculating the volume of Tyrode infused in the bladder to reach an intravesical pressure of 40 mm Hg it was measured together with the time required for the intravesical pressure and the flow rate of the pump (0.1 ml/min). The thresholds of firing of each afferent from inflamed bladders were calculated from the ramp of stimulation and compared with our database of afferents from normal bladders.

After several (3-4) stimulations to ensure that the afferent activity was stable, pharmacological assays were done on inflamed bladder. In a first series of experiments, a bolus (intravesical administration with the outlet valve opened) of a non selective cannabinoid agonist AZ12646915 (100 μl, 100 μM) dissolved in Tyrode + 1% DMSO was administered between two inflations, with 20 minutes prior to the second stimulation. This dose was selected as it was shown to be effective in reducing the afferent activity in our previous study. In addition, we observed a recovery and no effect of the vehicle [3]. To assess the specificity of action of AZ12646915, CB_1 _receptor antagonist AM251 (100 μl, 100 μM) or the CB_2 _receptor antagonist AM630 (100 μl, 100 μM) were administered 5 minutes prior AZ12646915 in another series of experiments using the same conditions of stimulation as above. Both AM251 and AM630 are selective antagonists with high affinity for CB_1 _and CB_2 _receptors respectively. The dose of AM251 selected has been shown to be efficient in blocking the effect of AZ12646915 in normal bladders [3], the same dose was selected for AM630 since AZ12646915 has equal affinity for CB_1 _and CB_2 _receptors.

Afferent activity was measured as the number of spikes/s counted at every 5 mm Hg intervals during each ramp of stimulation going from 0 to 40 mm Hg. Agonist and antagonists effects were measured using the normalized activity: each point of the ramps of pressure (pre and post-drug) was expressed as a percentage of the maximal activity recorded in the pre-drug stimulation.

### Immunofluorescence

A vascular rinse was performed by transcardial perfusion with 50 ml of PBS 0.1 M + 1 UI heparin/ml followed by 200 ml of PBS + 4% paraformaldehyde as a fixative solution. The bladder was harvested and placed in the same fixative solution during 3 hours at +4°C then transferred in PBS 0.1 M + 30% sucrose overnight at +4°C. After permeabilization (PBS, 10% Normal Goat Serum (NGS), 0.25% TRITON, 0.05% NaN_3_) and saturation with PBS + 20% NGS, cryostat sections (12 μm) of the bladder were incubated 48 h at +4°C with the primary antibodies solution: anti CB_1 _raised in rabbit and anti TRPV1 raised in guinea pig (Table 1). After rinsing, the sections were incubated with goat anti-rabbit IgG (1:600) conjugated with fluorescein isothiocyanate (FITC, 488 nm) and with goat anti guinea pig IgG (1:600) conjugated with Texas red (568 nm) 1 h at RT. Mounted slides were observed under a Zeiss Axioplan 2 Imaging fluorescence microscope and images were captured by a digital camera AxioCam HRc (Zeiss, Canada). Microphotographs were obtained with ×20 objectives. Negative controls were sections incubated with the primary antibodies preadsorbed with their respective blocking peptide (Table 1) and the time of exposure setting was constant during the acquisition of images. Background subtraction and merging of images were performed with the program Image J [54].

**Table 1:** Primary antibodies and their respective blocking peptides used for the immunofluorescence study.

Primary Antibodies	Catalog # (antibodies)	Host	Dilution	Epitope - Blocking peptide	Catalog # (blocking peptides)	Ratio (v/v) antibody/peptide	Source
**CB_1_**	10006590	Rabbit - Polyclonal	1:100	C-terminal MSVSTDTSAEAL	10006591	1:2	Cayman Chemical

**TRPV1**	GP14100	Guinea pig - Polyclonal	1:1000	C-terminal YTGSLKPEDAEVFKDSMVPGEK	P14100	1:5	Neuromics

### Chemicals

The cannabinoid compound used in this study, AZ12646915 (Astra Zeneca R&D, Montreal, Canada) has equal affinity for both human CB_1 _and CB_2 _receptor (Ki = 17 nM and 16 nM respectively). It was selected for its good solubility (up to 150 μM) in aqueous solution in order to prevent as much as possible precipitation in the tubing. The cannabinoid receptors antagonists AM251 and AM630 were purchased from Tocris Bioscience (Ellisville, USA). Cyclophosphamide was purchased from Sigma-Aldrich (Canada). Salts used for the solutions were all purchased from ACP chemicals (Montreal, Canada).

### Statistics

Differences between normal and inflamed animals were assessed with *t*-test for the behavior and Mann-Whitney test for spike rate. Effects of drugs were evaluated with paired *t*-tests (Wilcoxon matched pairs test). One sample *t*-test (versus a theoretical mean of 100) was used for the change of activity measured with the area under the curves for the antagonists study. *P *< 0.05 was considered statistically significant. Statistical analyses were performed using GraphPad Prism version 5.01 for Windows, GraphPad Software, San Diego California USA.

## List of abbreviations

CB_1_: receptor: cannabinoid receptor 1; CB_2_: receptor cannabinoid receptor 2; CGRP: calcitonin gene related peptide; CYP: cyclophosphamide; i.p.: intraperitoneal; TRPV1: transcient receptor potential vanilloid 1.

## Competing interests

The authors declare that they have no competing interests.

## Authors' contributions

J-SW and FC conceived and designed the experiments; J-SW performed the experiments; J-SW and FC wrote the manuscript. Both authors read and approved the final manuscript.
